# Feasibility of In-Home Sensor Monitoring to Detect Mild Cognitive Impairment in Aging Military Veterans: Prospective Observational Study

**DOI:** 10.2196/16371

**Published:** 2020-06-08

**Authors:** Adriana Seelye, Mira Isabelle Leese, Katherine Dorociak, Nicole Bouranis, Nora Mattek, Nicole Sharma, Zachary Beattie, Thomas Riley, Jonathan Lee, Kevin Cosgrove, Nicole Fleming, Jessica Klinger, John Ferguson, Greg John Lamberty, Jeffrey Kaye

**Affiliations:** 1 Minneapolis Veterans Affairs Health Care System Minneapolis, MN United States; 2 Department of Psychiatry University of Minnesota Minneapolis, MN United States; 3 Department of Neurology Oregon Health and Science University Portland, OR United States; 4 Oregon Center for Aging and Technology Portland, OR United States; 5 Division of Rehabilitation Science Department of Rehabilitation Medicine University of Minnesota Minneapolis, MN United States

**Keywords:** aging, mild cognitive impairment, activities of daily living, technology

## Abstract

**Background:**

Aging military veterans are an important and growing population who are at an elevated risk for developing mild cognitive impairment (MCI) and Alzheimer dementia, which emerge insidiously and progress gradually. Traditional clinic-based assessments are administered infrequently, making these visits less ideal to capture the earliest signals of cognitive and daily functioning decline in older adults.

**Objective:**

This study aimed to evaluate the feasibility of a novel ecologically valid assessment approach that integrates passive in-home and mobile technologies to assess instrumental activities of daily living (IADLs) that are not well captured by clinic-based assessment methods in an aging military veteran sample.

**Methods:**

Participants included 30 community-dwelling military veterans, classified as healthy controls (mean age 72.8, SD 4.9 years; n=15) or MCI (mean age 74.3, SD 6.0 years; n=15) using the Clinical Dementia Rating Scale. Participants were in relatively good health (mean modified Cumulative Illness Rating Scale score 23.1, SD 2.9) without evidence of depression (mean Geriatrics Depression Scale score 1.3, SD 1.6) or anxiety (mean generalized anxiety disorder questionnaire 1.3, SD 1.3) on self-report measures. Participants were clinically assessed at baseline and 12 months later with health and daily function questionnaires and neuropsychological testing. Daily computer use, medication taking, and physical activity and sleep data were collected via passive computer monitoring software, an instrumented pillbox, and a fitness tracker watch in participants’ environments for 12 months between clinical study visits.

**Results:**

Enrollment began in October 2018 and continued until the study groups were filled in January 2019. A total of 201 people called to participate following public posting and focused mailings. Most common exclusionary criteria included nonveteran status 11.4% (23/201), living too far from the study site 9.4% (19/201), and having exclusionary health concerns 17.9% (36/201). Five people have withdrawn from the study: 2 with unanticipated health conditions, 2 living in a vacation home for more than half of the year, and 1 who saw no direct benefit from the research study. At baseline, MCI participants had lower Montreal Cognitive Assessment (*P*<.001) and higher Functional Activities Questionnaire (*P*=.04) scores than healthy controls. Over seven months, research personnel visited participants’ homes a total of 73 times for technology maintenance. Technology maintenance visits were more prevalent for MCI participants (*P*=.04) than healthy controls.

**Conclusions:**

Installation and longitudinal deployment of a passive in-home IADL monitoring platform with an older adult military veteran sample was feasible. Knowledge gained from this pilot study will be used to help develop acceptable and effective home-based assessment tools that can be used to passively monitor cognition and daily functioning in older adult samples.

## Introduction

### Background

By year 2050, the number of people living with dementia is projected to triple to 115 million as the world’s aging population continues to grow rapidly [1,2]. There will be an increased demand for health care institutions and researchers to respond and develop preventative strategies to address the growing needs of the aging population. An important subgroup of the larger aging population is aging military veterans. Aging military veterans are at an elevated risk for developing dementia because of their unique military histories (eg, traumatic brain injury and posttraumatic stress disorder) [3-6] and their increased incidence of vascular risk factors (eg, diabetes, hypertension, and hyperlipidemia) [7-9]. In 2017, the prevalence of Alzheimer disease (AD) among US military veterans was 750,000, an increase of 166% from 2014 [10]. On average, the expense related to lifetime dementia care is US $350,174 (in 2018) per person, which is US $150,303 more than the expense for those without dementia [11]. As neurodegenerative disorders such as AD progress slowly and over a long period of time [12], early detection of dementia is crucial and has the potential to reduce the number of individuals and caregivers affected by the disease [10]. Identifying individuals with mild cognitive impairment (MCI), which often represents the prodromal stage of several neurodegenerative diseases (including AD) [13], could lead to targeted interventions that ultimately improve daily function and independence.

Although basic activities of daily living such as bathing, grooming, and eating are affected later in the course of neurodegenerative diseases after cognitive impairment has progressed and significantly impacted daily functions, subtle difficulties performing instrumental activities of daily living (IADLs) are present earlier in the course of disease. These IADLs are behavioral signs that may signal the progression from normal aging to MCI providing the opportunity for early detection and intervention [14-19]. IADLs are cognitively complex daily activities that require multiple constituent cognitive processes to perform accurately and efficiently and that are crucial for independent living. Examples of IADLs include managing medications and finances, driving a motor vehicle, and using every day technology such as computers and mobile phones [20]. However, early detection measures for MCI currently face many challenges and fail to identify change in real time because of infrequent episodic clinic visits [21]. The sensitivity and specificity of cognitive screeners used to detect impaired cognition has an unacceptable proportion of false positives and negatives and fails to approximate a patient’s real-world difficulties. In addition, patient self-report and collateral information can be biased or unreliable because of worry, stress, or forgetfulness [21,22]. Therefore, a comprehensive neuropsychological battery has been considered the standard for clarifying the nature and extent of someone’s cognitive deficits [23]. Unfortunately, these neuropsychological evaluations are time-consuming, expensive, do not provide a clear appraisal of one’s functional performance, and are not widely accessible to older adults who face socioeconomic and geographic barriers to specialty care services.

An alternative assessment approach to capture changes in cognitive status is the unobtrusive collection of continuous activity data over long periods of time [21,22]. Real-world assessment technologies have allowed researchers to continuously monitor cognitively demanding functional activities in one’s home environment to identify abnormal activity patterns predictive of MCI [22,24,25]. Everyday consumer devices (eg, medication pillbox, and home computer) are being used to continuously observe cognitively challenging IADLs. This offers a practical, low cost, and noninvasive approach to assessing changes in one’s daily functioning [19,26].

### This Study and Objectives

Despite a growing interest in the use of real-world assessment approaches to detect early signs of MCI, there are still many gaps in the current literature. This paper has described a 12-month pilot study, Promote Independent Aging (PRIA) is among the first to deploy unobtrusive sensor-based assessment technologies in the homes of aging military veterans in the community. PRIA partners with the Oregon Center for Aging and Technology (ORCATECH) [21] and the Collaborative Aging Research using Technology (CART) initiative [27], utilizing components of the ORCATECH-CART in-home and mobile sensor assessment platform and infrastructure to identify and monitor meaningful changes in routine daily activities that are affected by MCI, such as computer use, medication taking, physical activity, and sleep.

In this paper, we have described the sample of participants recruited into the study, the clinical assessment procedures, and the in-home sensor-based assessment platform and monitoring of the activity measures (outcome variables) in the study. We have discussed our experiences with feasibility (eg, recruitment, retention, installation, and in-home technology maintenance visits) of the in-home monitoring technologies. Finally, we have concluded with a discussion about future directions, limitations, and clinical implications of this research.

## Methods

### Study Participants

Participants were 30 community-dwelling older adult military veterans. Of this group, 15 were classified as healthy controls and 15 were classified as MCI using established clinical and research measures. All participants were recruited from the Minneapolis-Saint Paul, Minnesota metropolitan area, and gave written informed consent before participating in study activities. The protocol was approved by the Minneapolis Veterans Affairs Health Care System’s (MVAHCSs) institutional review board (IRB #4748-A). Enrollment began in October 2018 and continued on a rolling basis until January 2019. Participants were compensated US $40 per month over the course of 12 months. See Textbox 1 for participant inclusion criteria.

Participants were recruited through recruitment letters and advertising (eg, flyers) targeted toward patients seen at the MVAHCS in primary care and specialty clinics serving older adult military veterans. Initial screening data were also pulled from the Veterans Affairs (VA) Informatics and Computing Infrastructure and the computerized patient record system to screen military veterans for eligibility based on age, location, and health history. Recruitment letters were sent out to patients who met basic eligibility criteria from these datasets, and follow-up calls were made within 2 weeks to interested parties. Potential candidates were also pulled from a clinical database that includes neuropsychological, clinical, and demographic information from over 2000 military veterans referred for outpatient neuropsychological evaluations since 2014 at the MVAHCS (VA IRB#: 4637-B).

**Promote Independent Aging inclusion criteria.**

- Aged 65 years or older
- Live within 30 miles of the Minneapolis Veterans Affairs Health Care System
- Live independently in their home (living with a companion or spouse was allowed but not as a caregiver)
- Take at least one medication daily and willing to use the instrumented study pillbox
- Have a home internet connection
- Own a computer and use it at least once per week
- Relatively healthy for age (no poorly controlled or unstable medical conditions or major neurological disorders)
- Absence of moderate to severe depression (Geriatric Depression Scale score-15≥7) [28]
- Absence of moderate to severe anxiety (Generalized anxiety disorder-7 questionnaire score>5) [29]
- No impaired global cognition (Montreal Cognitive Assessment sex, age, and education adjusted z-scores<−2) [30]
- Do not meet criteria for dementia (having a global Clinical Dementia Rating Scale score of less than or equal to 0.5 indicating no major impairment in daily functioning) [31]

### Clinical Assessment Procedures

Participants were assessed at the MVAHCS Geriatric Research, Education and Clinical Center at baseline and 12 months. Research staff met with participants as well as their study informant (usually a spouse, close family member, or friend) during the baseline and final study visit. A battery of standardized neuropsychological tests, health assessments, and daily function questionnaires (eg, Functional Activities Questionnaire [32]) was administered (see Table 1), with a subset of this assessment battery from the Uniform Data Set of the National Alzheimer’s Disease Coordinating Center [33] in addition to other well-validated measures used in prospective National Institute on Aging (NIA)–funded longitudinal aging cohort studies. Health assessments consisted of a review of medical histories, medication lists, and completion of the Modified Cumulative Illness Rating Scale (MCIRS) [34,35]. The neuropsychological examination included the following battery of well-established and validated tests assessing multiple cognitive domains: attention and processing speed (Number Span Forward, Trail Making Part A, Stroop Color Naming, Stroop Word Reading) [33,36,37], working memory (Number Span Backward) [33], Memory (Craft Story Recall; Consortium to Establish a Registry for Alzheimer’s Disease delayed recall and recognition; and Benson Complex Figure Delayed Recall) [38-40], language (Multilingual Naming Test and Category Fluency) [39,41,42], executive functioning (Stroop Color-Word; Verbal Fluency; Trail Making Part B) [33,36,37], and visuospatial construction (Benson Complex Figure Copy) [40].

**Table 1:** Promote Independent Aging study visit information.

Assessment	Baseline visit	Final study visit (month 12)
Consent and authorization forms	+^a^	N/A^b^
Demographics form	+	N/A
Socioeconomic and employment form	+	+
Physical assessment form	+	+
Mobility form	+	+
Personal and family health history	+	+
Modified Cumulative Illness Rating Scale [33,34]	+	+
Physical Activity Scale for the Elderly [43]	+	+
Pittsburg Sleep Quality Index [44]	+	+
Older Americans Resources and Services activities of daily living and IADL^c^ [45]	+	+
Everyday Cognition Questionnaire self and informant [46,47]	+	+
Functional Activities Questionnaire [31]	+	+
Clinical Dementia Rating self and informant [30]	+	+
Habits form	+	+
Cognitive status form	+	+
Montreal Cognitive Assessment [29]	+	+
Neuropsychological examination (see text)	+	+
Generalized anxiety disorder 7-item [28]	+	+
Geriatric Depression Scale-15 item [27]	+	+
University of California, Los Angeles Loneliness Scale [48]	+	+
Lubben Social Network Scale [49,50]	+	+
RAND 36-Item Health Survey [51]	+	+

^a^+: measure is administered.

^b^N/A: not applicable.

^c^IADL: instrumental activities of daily living.

### In-Home Activity Monitoring Platform and Installation

Daily activity data were collected using a well-established unobtrusive in-home activity assessment system installed in the home of each study participant. The in-home assessment platform is developed and managed by the ORCATECH [21]. ORCATECH is a National Institutes of Health (NIH)/NIA–funded research center that develops, implements, and supports leading-edge technologies for clinical research. The specific devices used in the study were chosen because they are included in the NIA-funded ORCATECH in-home technology assessment platform and are compatible with the ORCATECH technology infrastructure. This research platform is currently widely deployed across the United States as part of the VA and NIH CART initiative. In this study, the ORCATECH platform installation occurred within 4 weeks of the participant’s baseline study visit and took place at the participant’s home. Aside from the brief Web-based surveys, the devices used in this study gathered information from participants passively and did not require training or new learning. The pillbox used in the study was a standard 7-day pillbox familiar to participants and did not require formal instruction. Participants were current computer users at study entry, and no training was required for using their personal computers or completing web-based surveys. The activity tracker watch required no training, as it was worn like a regular watch. The passive nature of the data collection is critical to feasibility and long-term retention because of low participant effort and burden. Study devices (such as, pillbox, watch, and computer software, described in detail in the following sections) were purchased and maintained by ORCATECH and installed by VA research personnel. VA and ORCATECH research personnel monitored technology through the ORCATECH Management Console interface on a weekly basis to ensure that all devices were working properly. In the event of technical difficulty, research personnel repaired or replaced technology within 1 to 2 weeks. At the end of the 12 months, ORCATECH study devices were removed from participants’ homes.

#### Hub Computer (Raspberry Pi 3 Model B, Pencoad, Wales)

The hub computer [52] received and transferred all deidentified sensor data collected at the participant’s home (medication taking and fitness tracker) via a secure virtual private network (VPN) connection to the secure ORCATECH research server. The hub computer, which is placed unobtrusively in the home, broadcasts a wireless network in the participant’s home, acts as a client to a wireless or wired router, and checks in with the ORCATECH server to ensure that the in-home monitoring devices are up to date and properly identified. The in-home activity data were sent from the hub computer to the ORCATECH server on a continuous basis and was deleted from the device afterwards.

#### Medication Tracking Pillbox (TimerCap iSort, Moorpark, CA)

The pillbox [53] recorded timestamps of when the lids of the 7-day pillbox (one lid for each day of the week) were opened or closed and transmitted the information to the hub computer via Bluetooth Low Energy (BLE). The pillbox recorded whether or not the compartment was opened (and closed) and the time or times of day that it was opened. If the pillbox did not have a connection to the hub computer, data were cached locally to the device until the next successful connection. The pillbox caches 2 to 3 weeks of data. Data were transmitted securely to servers at ORCATECH via a VPN, and pillboxes were linked to participant ID numbers.

#### Wrist-Worn Fitness Tracker (Nokia Steel, Issy-les-Moulineaux, France)

The wrist-worn wearable device [54] collected physical activity data (ie, steps taken and time spent sleeping) and transmitted information to the hub computer via BLE. This device communicates data acquired several times a day to the hub computer. If the wearable device was not able to connect to the hub computer, data were cached locally to the device until the next successful connection. The wearable device caches 3 weeks of data. Data were transmitted securely to servers at ORCATECH via a VPN.

#### Computer Use Monitoring Software (Worktime Corporate, Woodbridge, Ontario)

Computer use monitoring software was installed on participants’ own computers by VA research personnel. This commercially available software collected information about number and duration of computer sessions such as log-in/log-off times, active/idle times, and time spent on types of applications (internet and documents). Advanced Encryption Standard encrypted data (FIPS 140-2 compliant) was transmitted to ORCATECH servers via Transmission Control Protocol connection. Document, names, and Web URLs were excluded from monitoring. Worktime Corporate [55] does not record keystrokes, passwords, emails, chats, document content, or screen content. This computer software is only compatible with Windows 7, 8, 8.1, and 10. The computer software is not compatible with Mac operating system; 8 participants with Mac computers did not have Worktime Corporate installed on their personal computers. Thus, 8 participants did not have computer metrics measured in this study.

#### Web-Based Health Update Questionnaire (5-10 Min Per Week)

Participants received a brief, weekly Web-based 13-item health questionnaire (see Multimedia Appendix 1) that asked questions about events and behaviors that could affect in-home monitoring activity patterns (eg, medication changes, falls, injuries, health changes, emergency room visits, depression, changes to living space, vacations, and visitors) [21,56]. This survey was administered via the Qualtrics Survey Platform [57] and sent through email every Monday at 9 AM (Central Time [CT]). If a participant failed to complete the survey by Wednesday of each week, another survey was sent automatically on Wednesday at 9 AM (CT). If the participant failed to complete the follow-up survey by Thursday or Friday of each week, phone calls were made to each participant to ensure data capture and quality.

#### Survey for Memory Attention and Reaction Time (5-10 Min Per Month)

Participants received a monthly Web-based memory test (see Multimedia Appendix 2) called the Survey for Memory Attention and Reaction Time (SMART) [58], which included four short cognitive tasks (including versions of the Trail Making Test B and the Stroop Color-Word Interference tasks). The SMART survey was administered via the Qualtrics Survey Platform and was sent on the last Monday of each month at 9 AM (CT). If a participant failed to complete the survey by Wednesday of each week, another survey was sent automatically on Wednesday at 9 AM (CT). If the participant failed to complete the follow-up survey by Thursday or Friday of each week, phone calls were made to each participant to ensure data capture and quality.

### Statistical Analyses

Recruitment and retention numbers, baseline demographic and clinical characteristics, and common technological difficulties are presented for the overall cohort as well as by study group (MCI vs intact cognition). Differences between the two study groups were assessed using 2-tailed *t* tests or the Wilcoxon rank sum test for continuous variables (depending on the distribution) or by using the Pearson chi-square test or Fisher exact test for categorical variables (depending on cell size). All summaries and analyses were performed using SPSS version 24.

## Results

### Participant, Recruitment, and Retention

PRIA research personnel received 201 calls to participate. A total of 150 people were screened by research personnel. People were not screened either because they did not return research personnel phone calls or because people called after study slots were filled. Of the 150 screened, the most common exclusion criteria included nonveteran status (17/150, 11.3%), living too far from the study site (14/150, 9.3%), and having exclusionary physical or mental health concerns (27/150, 18.0%). Of those enrolled, 35 participants had the full clinical assessment, and 32 participants had the research technology platform installed in their home. On average, the MCI group had 185.3 (SD 27.1; n=15) days of follow-up (range 113-213) and the cognitively intact group had 150.9 (SD 33.8; n=15) days of follow-up in a 7-month monitoring period (range 63-205).

Following baseline evaluation, 5 participants withdrew from the study. Specifically, 2 participants withdrew before installation of the technology because they were away from the metropolitan area for half of the year without an internet connection, and 1 participant withdrew because they saw no direct benefit from this research study. Two participants withdrew following technology installation because of unanticipated acute medical events. A summary of demographic and clinical characteristics of the final sample (N=30) is presented in Table 2.

**Table 2:** Participant baseline demographics and clinical characteristics (N=30).

Variable		Total	Mild cognitive impairment (n=15)	Healthy controls (n=15)	*P* value
Age at baseline (years), mean (SD)		73.5 (5.4)	74.3 (6.0)	72.8 (4.9)	.46
Sex (male), n (%)		28 (93)	14 (93)	14 (93)	N/A^a^
Race (white), n (%)		30 (100)	15 (100)	15 (100)	N/A
Education (years), mean (SD)		14.9 (2.0)	14.9 (2.3)	15.0 (1.9)	.86
Montreal Cognitive Assessment [29], mean (SD)		24.5 (2.3)	22.9 (1.8)	26.1 (1.5)	<.001
Geriatric Depression Scale [27], mean (SD)		1.3 (1.3)	1.5 (1.5)	1.1 (1.1)	.42
Generalized anxiety disorder [28], mean (SD)		1.3 (1.6)	1.7 (1.7)	0.9 (1.4)	.16
Functional Activities Questionnaire [31], mean (SD)		1.0 (1.6)	1.6 (1.8)	0.4 (1.1)	.04
Modified Cumulative Illness Rating Scale [33,34], mean (SD)		23.1 (2.9)	24.0 (3.3)	22.1 (2.2)	.08
Pittsburg Sleep Quality Index [44], mean (SD)		5.7 (3.2)	6.5 (3.7)	5.0 (2.4)	.21
Everyday Cognition Questionnaire (ECog) informant [18,46,47], mean (SD)		1.3 (0.3)	1.3 (0.4)	1.2 (0.3)	.39
ECog participant [18,46,47], mean (SD)		1.4 (0.5)	1.5 (0.7)	1.3 (0.2)	.17
Physical Activity Scale for the Elderly [43], mean (SD)		157.8 (57.9)	172.3 (66.5)	143.3 (45.5)	.18
**Health comorbidities (positive for the condition), n (%)**					
	Atrial fibrillation	4 (13)	4 (27)	0 (0)	.03
	Diabetes	5 (17)	2 (13)	3 (20)	.62
	Hypertension	19 (63)	12 (80)	7 (47)	.06
	Hypercholesterolemia	24 (80)	13 (87)	11 (73)	.36
	Sleep apnea	17 (57)	9 (60)	8 (53)	.71

^a^N/A: not applicable.

### Common Technical Difficulties

#### Hub Computer (Raspberry Pi 3 Model B)

The most common technical difficulty associated with the hub computer was the loss of connection to the hub computer, requiring research personnel to update the hub computer remotely. However, sometimes these updates needed to be done manually. For example, an unexpected license expiration lead to a temporary VPN handshake failure, which affected all 30 participants and delayed data capture for some participants up to 1 month. This VPN handshake failure could not be fixed remotely, and research personnel were required to update the hub computer manually across all 30 homes. Over a 7-month monitoring period, 13% (4/30) of participants in the entire sample required two or more in-home technology maintenance visits to repair the hub computer; all 4 were MCI participants, *P*=.10 (see Table 3).

**Table 3:** Technology device repairs and reminder phone calls over a 7-month monitoring period (N=30).

Variable	Total sample	Mild cognitive impairment (n=15)	Healthy controls (n=15)	*P* value
Total device repair visits, mean (SD)	2.4 (1.7)	3.1 (1.9)	1.8 (1.2)	.04^a^
Participants requiring >2 hub computer visit, n (%)	4 (13)	4 (27)	0 (0)	.10
Participants requiring >1 pillbox visit, n (%)	7 (23)	6 (40)	1 (7)	.08
Participants requiring >1 watch visit, n (%)	8 (27)	5 (33)	3 (20)	.68
Participants requiring >1 worktime visit, n (%)	11 (37)	6 (40)	5 (33)	>.99
Total reminder phone calls, mean (SD)	2.7 (2.9)	2.9 (3.2)	2.5 (2.8)	.67
Participants requiring >1 Web-based health questionnaire reminder call, n (%)	19 (63)	12 (80)	7 (47)	.13
Participants requiring >1 Survey for Memory Attention and Reaction Time survey reminder call, n (%)	18 (60)	8 (53)	10 (67)	.71

^a^Group comparisons were made using independent *t* tests for continuous variables or Fisher exact tests (2-tailed) for categorical variables.

#### Medication Tracking Pillbox (TimerCap iSort)

The most common technical difficulties associated with the pillbox included battery issues (which required a battery change) and batteries falling out of the pillbox (which could be prevented by taping the battery door shut with a piece of masking tape). Furthermore, broken compartment lids and broken contact pieces within the instrumented pillbox required research personnel to replace the lids or the pillbox altogether. Finally, there were issues associated with syncing the pillbox to the hub computer, which required research personnel to reset the Pi’s BLE connection. Over a 7-month monitoring period, 23% (7/30) of participants in the entire sample required one or more in-home technology maintenance visits to repair the instrumented pillbox; 6 were MCI participants, and 1 was a healthy control participant; *P*=.08 (see Table 3).

#### Wrist-Worn Fitness Tracker Watch (Nokia Steel)

The most common technical difficulties associated with the watch included low batteries within the watch, which required a battery change (batteries should last at least six months) as well as broken sensors within the watch, which required research personnel to replace the watch altogether. Furthermore, three watch faces were broken, which required research personnel to replace the watch. Finally, there were issues associated with synchronizing the watch to the hub computer, which required research personnel to reset the Pi’s BLE connection. Over a 7-month monitoring period, 27% (8/30) of participants in the entire sample required one or more in-home technology maintenance visits to repair the fitness tracker watch: 5 were MCI participants and 3 were healthy control participants; *P*=.68 (see Table 3).

#### Computer Use Monitoring Software (Worktime Corporate)

The most common technical difficulties associated with the computer use monitoring software was the removal of Worktime Corporate by malware detection and prevention programs installed on the participant’s computer requiring research personnel to redownload the software on participants’ computers. Furthermore, it came to our attention that Worktime Corporate was not compatible with certain versions of Mac computers as well as tablets or mobile phones. Thus, Worktime Corporate was only installed in 22 homes because 8 participants owned Mac devices. Two participants were married and shared the same computer with separate log-on accounts. Over a 7-month monitoring period, 36% (11/30) participants in the entire sample required one or more in-home technology maintenance visits to repair Worktime software: 6 were MCI participants and 5 were healthy control participants; *P*>.99 (see Table 3).

#### Web-Based Health Update Questionnaire

The most common technical difficulties associated with the Web-based health update questionnaire was that the email containing a link to the questionnaire would sometimes go to a participant’s Spam folder. This issue was mitigated by asking participants to save the email address in their contacts (or showing them how to do so). Furthermore, some research participants would complete the survey all the way through and then receive a message that they had failed to complete the survey. This would require participants to fill out the same survey twice. If participants failed to fill out their questionnaire by Thursday of each week, they would receive a reminder phone call from research personnel. Over a 7-month monitoring period, 63% (19/30) of participants in the entire sample required one or more reminder phone calls to complete the Web-based health questionnaire: 12 were MCI participants and 9 were healthy control participants; *P*=.13 (see Table 3).

#### Survey for Memory Attention and Reaction Time

The most common technical difficulties associated with the SMART survey was screen freezing. The freezing during a participant’s session was most often related to a portion of code that transferred mouse activity data back to the ORCATECH servers. Research staff discovered that the transfers were being called multiple times on each task and eventually overloaded the browser with unnecessary transfers. This issue was mitigated by having a programmer apply logic to check if there were transfers in progress, which ultimately reduced the frequency of transfers. Furthermore, although the SMART survey was compatible with every internet browser, operating system, and device, participants had trouble completing the SMART survey on their mobile phone. A total of 24 people completed the SMART survey on a computer, 4 participants used their mobile phone to complete the survey, and 2 participants used their tablet. Of note, the 2 tablet users were in the MCI group and 3 of the 4 mobile phone users were in the MCI group. Over a 7-month monitoring period, 60% (18/30) of participants in the entire sample required one or more reminder phone calls to complete the Web-based SMART survey: 8 were MCI participants and 10 were healthy control participants; *P*=.71 (see Table 3).

## Discussion

### Principal Findings

The results in this study demonstrate the feasibility of engaging older adult military veterans in an observational research study using in-home IADL monitoring technology. Recruitment was successful as evidenced by a high number of phone calls received from older adults to participate and study enrollment completed within 6 months. Retention was high; 86% (26/30) of individuals who were initially consented were retained 7 months into the study follow-up period. Five military veterans dropped out from the study; only one dropped out because of study-specific concerns. The 2 individuals who withdrew because of unanticipated medical events expressed interest in remaining in the study and continue contributing. Furthermore, frequency of in-home technology maintenance visits was relatively low across the sample given the duration of monitoring follow-up, as shown in Table 3. Overall, technology maintenance visits were more prevalent for MCI participants (*P*=.04) than healthy controls, although reminder telephone calls to complete Web-based surveys were not (*P*=.67). One possible explanation for this finding is that when the devices (eg, watch and pillbox) in the study lost internet connection with the hub computer because of software or firmware updates, healthy control participants were better able to successfully troubleshoot and problem solve technological difficulties remotely over the phone with research staff compared with MCI participants, preventing research staff from making additional trips to repair devices. In contrast, because of their MCIs, MCI participants may have been less successful in understanding and retaining complex instructions over the phone and organizing and executing device repairs or resets requiring multiple steps without in-person assistance. Troubleshooting device and internet connection issues is a cognitively complex task that requires executive functions, memory, language, visual spatial abilities, and processing speed. Compared with the devices used in the study, the Web-based surveys required less troubleshooting by participants. In general, once participants initiated taking the Web-based surveys by clicking a link in their email, the survey software program worked well. Our results indicate/suggest that the sample of MCI participants remembered to take the Web-based surveys as reliably as healthy controls, but they had more difficulty carrying out cognitively complex tasks with higher executive demands such as troubleshooting device resets and repairs as well as healthy controls.

Various factors could be related to the high rates of retention and participants’ motivation to participate in this kind of research. First, real-world assessment research allows for rapport and relationship-building between research staff and study participants from the onset of the study. Real-world assessment research requires the installation of technology in the participants’ home, as compared with scenario-based assessment research where the interaction occurs in a clinic-based setting [24]. Research staff are welcomed into the homes of aging military veterans and able to develop fulfilling relationships with research participants. Real-world assessment research also facilitates a deeper understanding about a person’s needs and daily functioning compared with conducting a clinical interview in a clinic-based setting. Developing this unique rapport/relationship with research participants started at the baseline study visit where research staff met with study participants and their study informant (usually a spouse, close family member, or friend). The information acquired from the participant and informant during the study visit created a holistic picture of each participant, allowing research personnel to generate a multifaceted view of each person. Measures such as the CDR [31] and PASE [43] include questions about the participant’s hobbies, physical activities, family, and about recent events that happen in their lives. These questions allowed research personnel to connect to the study informant and the study participant and develop trust and familiarity before they entered the participant’s home.

### Strengths and Limitations

The overall experience with the ORCATECH platform was positive. Research technical staff were able to learn quickly how to install and trouble shoot technology, and research participants were generally receptive to the technology. However, as is common when working with diverse technologies, there were some limitations particularly with commercially available devices and software integrated into the ORCATECH platform. For example, 8 older adults in our study have macOS-based computers so Worktime Corporate [55] could not be installed, thus preventing usage data capture. Furthermore, as Worktime Corporate can only be installed on traditional computers, we failed to capture information on participant’s mobile phones or tablets. In future studies, monitoring software should be developed that is compatible across all platforms, operating systems, and devices so that all data are captured. Other limitations include the durability of the commercially available devices used in the study. The pillboxes had limited durability, which required research personnel to replace pillbox lids and pillboxes frequently. Furthermore, watches had to be replaced because of broken watch faces (3/30, 10% of participants over a span of 7 months). In contrast, across other ORCATECH studies (including CART) involving over 200 primarily nonveteran, female aging participants, 2.0% (5/250) participants have broken the face of their watch over a span of multiple years. Other limitations included the homogeneity of our sample (30/30, 100% white; 28/30, 93% male), non-inclusion of military veterans who lived outside of the metropolitan area (eg, rural populations), and noninclusion of military veterans who lived in another home during the winter (*snow birds)*. This study was funded by the Veterans Health Administration, which required recruitment of military veterans only. The older adult military veteran population in Minnesota, where the study was conducted, is largely represented by white men [59]. Future studies will expand recruitment to nonveteran samples that are more heterogeneous and balanced regarding gender and race. Despite the homogeneity of our cohort, there was diversity within this sample regarding the cohort’s varying health comorbidities (mean MCIRS score 23.1, SD 2.9) and educational attainment. Duration of education ranged from 9 to 18 years (mean education 14.9, SD 2.0). Other in-home technology and aging studies report higher educational levels and lower MCIRS levels in their cohorts; thus, this research helps to increase our knowledge about the feasibility of in-home technologies in a wider array of older adults [16,19,21].

### Future Directions and Conclusions

Future analyses will establish which sensor monitored IADL variables best discriminate between MCI and healthy controls and will explore trajectories of IADL functioning in MCI and healthy controls. Few studies [22,60] discuss the acceptability of the technology used over time, especially from the participant’s perspective. Future directions will include deployment of an in-home monitoring technology perception survey to the military veterans in this cohort. This survey will allow us to capture participants’ perceptions about the in-home technology they have been using in the study as well as their perceptions about how they would want to use in-home monitoring technology in the future. Furthermore, very few studies [24] have explored ethics related to in-home monitoring which is important to discuss to implement these technologies in people’s homes. This survey will explore ethical issues associated with real-world assessment research such as privacy, security concerns, and who should have access to the data. This will give us a better idea of how comfortable older adult military veterans feel with the usage of their activity data in the future. Other future directions include using this technology to extend beyond IADL and cognitive assessment to help identify people at risk for acute medical events (stroke and infections). This research study also was able to install technology and monitor a participant’s activity in a rehabilitation center following an unanticipated medical event (data not shown), demonstrating the flexibility of the methodology. Future studies should investigate the feasibility of incorporating these methodologies in a variety of health care settings outside of the home, including inpatient medical facilities when higher levels of care for individuals are temporarily required. Future studies using activity monitoring technologies to detect and monitor cognitive decline should aim to increase inclusion of individuals who are typically underrepresented in aging and dementia research such as younger older adults, people of color, adults living in rural communities, and individuals with low socioeconomic status.

Knowledge gained from this pilot study will be used to help develop acceptable and effective home-based assessment tools that can be used within the VA system to monitor cognition and daily functioning in aging military veterans. The results and lessons provided by this study have importantly been incorporated into improving the national CART initiative platform, which has now been deployed to diverse cohorts of older adults including rural-residing military veterans.

## Appendix

Multimedia Appendix 1 Sample questions for the web-based health update questionnaire.

Multimedia Appendix 2 Opening page of the Survey for Memory, Attention, Responding, and Thinking.

## Abbreviations

AD: Alzheimer disease
BLE: Bluetooth Low Energy
CART: Collaborative Aging Research using Technology
CT: Central Time
IADL: instrumental activities of daily living
IRB: institutional review board
MCI: mild cognitive impairment
MCIRS: Modified Cumulative Illness Rating Scale
MVAHCS: Minneapolis Veterans Affairs Health Care System
NIA: National Institute on Aging
NIH: National Institutes of Health
ORCATECH: Oregon Center for Aging and Technology
PRIA: Promote Independent Aging
SMART: Survey for Memory Attention and Reaction Time
VA: Veterans Affairs
VPN: virtual private network
