# Supplementary figures and images for: Feasibility of In-Home Sensor Monitoring to Detect Mild Cognitive Impairment in Aging Military Veterans: Prospective Observational Study
Source: JMIR Form Res. 2020 Jun 8;4(6):e16371. doi: 10.2196/16371 (PMC7308933; doi:10.2196/16371)

**
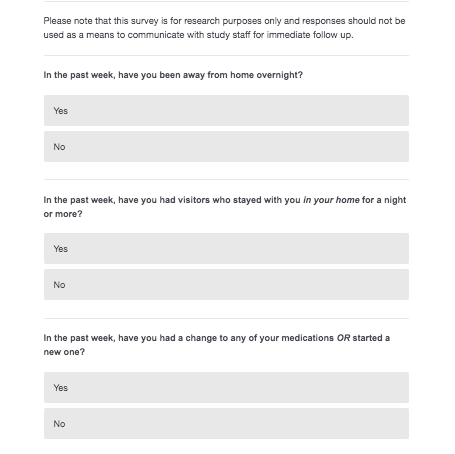
**

Supplement: Multimedia Appendix 1 [file formative_v4i6e16371_app1.docx]

**
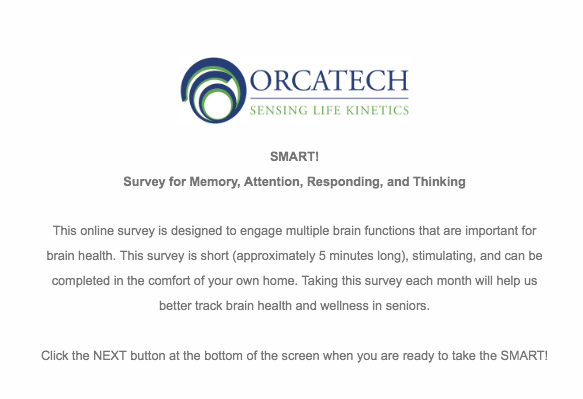
**

Supplement: Multimedia Appendix 2 [file formative_v4i6e16371_app2.docx]
